# Composition of Carotenoids and Flavonoids in Narcissus Cultivars and their Relationship with Flower Color

**DOI:** 10.1371/journal.pone.0142074

**Published:** 2015-11-04

**Authors:** Xin Li, Min Lu, Dongqin Tang, Yimin Shi

**Affiliations:** School of Agriculture and Biology, Shanghai Jiao Tong University, Shanghai, China; UCLM, SPAIN

## Abstract

Narcissus is widely used for cut flowers and potted plants, and is one of the most important commercial bulbous flowers in the floricultural industry. In this study, ten carotenoid and eighteen flavonoid compounds from the perianths and coronas of fifteen narcissus cultivars were measured by HPLC–APCI-MS/MS and UPLC-Q-TOF-MS/MS. Among these, six carotenoids, a total of seventeen flavonols and chlorogenic acid were identified in narcissus for the first time. A multivariate analysis was used to explore the relationship between flower color and pigment composition. We found that all-*trans*-violaxanthin and total carotenoid content were the main factors that affected flower color. These investigations could provide a global view of flower color formation and a theoretical basis for hybridization breeding in narcissus.

## Introduction

Narcissus, a flowering plant of the Amaryllidaceae family, is a typical Mediterranean genus of geophyte, with between 60 and 80 separate species [1, 2].Narcissus species cover a wide variety of habitats, and they range from narrow-range endemic to much more widespread species. In recent years, growing attention has been focused on the narcissus family, owing mainly to its unique flower shape (cup-shaped or crown-like corona) and outstanding flower color.

**Fig 1:**
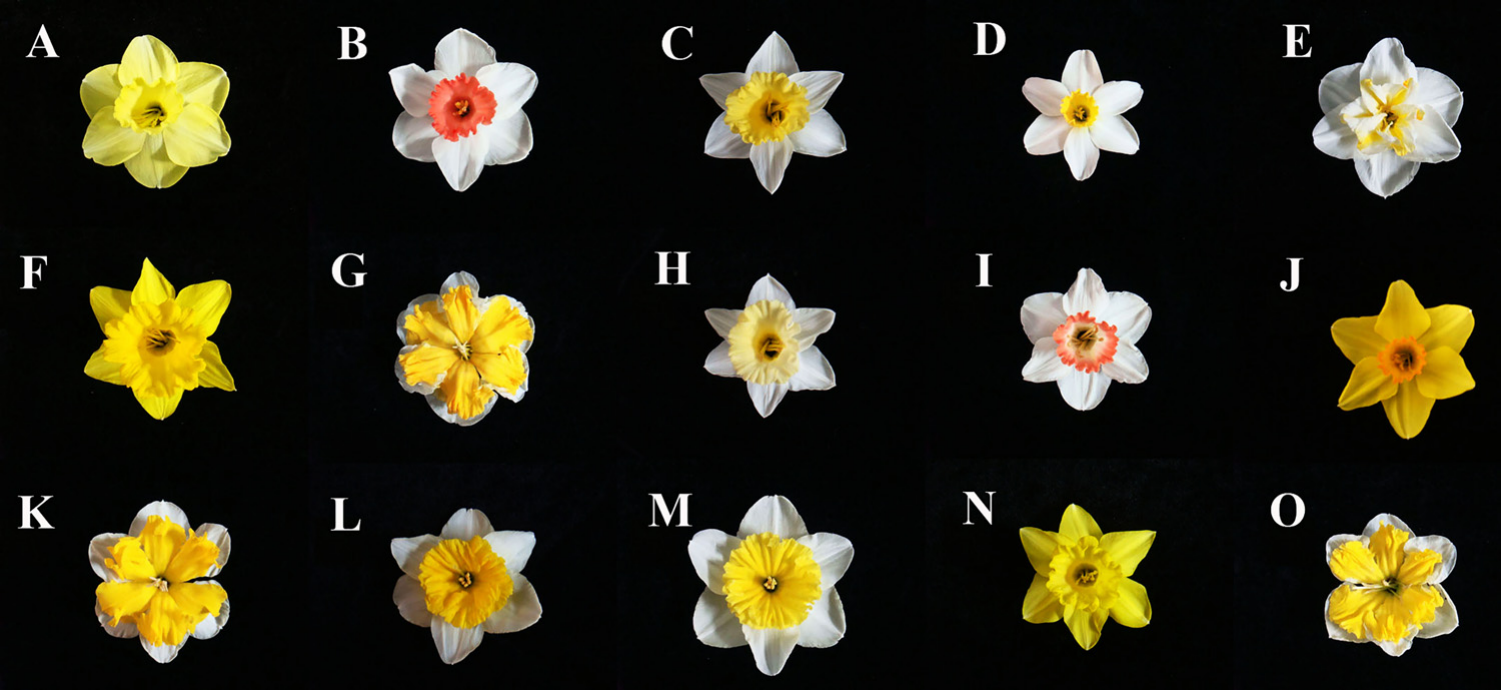
Flowers of narcissus cultivars: avalon (A), decoy (B), gigantic star mutation (C), jack snipe (D), lemon beauty (E), marieke (F), mondragon (G), mount hood (H), pink charm (I), pinza mutation (J), shangnong dieying (K), shangnong ruhuang (L), slim whitman (M), spellbinder (N), valdrome (O).

Flower pigmentation is determined by the synthesis and accumulation of plant secondary metabolites such as carotenoids, flavonoids and betalains [3]. Many studies have explored the relationship between flower color and pigment composition [4–6].In flowers, carotenoids synthesized in the chromoplasts are responsible for petal colors in the yellow-to-orange range [7,8]. Several investigations of the carotenoid composition of petals have been reported. These studies suggest that carotenoids in petals have distinctive compositions that are species-dependent [9,10]. Flavonoids have been extensively studied in many kinds of plants, and they are the decisive pigment presented in some flower colors.[6,11]In addition to their contribution to flower color, flavonoids have potential health-promoting benefits in humans including antioxidant, anti-inflammatory and anti-cancer properties [12,13]. As shown in Fig 1, narcissus flowers may be colored pink, white, yellow and/or orange; these colors are attributed to flavonoids and carotenoids.

To our knowledge, with the exception of some limited reports [14–16] there have been no detailed studies which have investigated and characterized the composition of carotenoid and flavonoid in flower pigment composition in different kinds of narcissus. Furthermore, the formation and genetic mechanisms underlying flower color in narcissus have been unclear. In this study, we investigated the carotenoid and flavonoid compositions of fifteen narcissus cultivars from their perianths and coronas using HPLC-APCI-MS/MS and UPLC-ESI-MS/MS, which are robust methods for the separation and identification of carotenoids and flavonoids respectively. The results of this analysis allowed us to undertake the first preliminary description of the genetic mechanisms responsible for the flower color in narcissus. Additionally, the relationship between flower color and pigment composition was also studied. This study therefore describes in detail the chemistry underlying pigment formation in narcissus, and provides information for future breeding studies.

## Materials and Methods

### Standards and solvents

Standards of all-*trans*-neoxanthin, all-*trans*-violaxanthin, all-*trans*-*β*-carotene, all-*trans*-lutein all-*trans*-β-cryptoxanthin and quercetin 3-*O*-rutinoside (rutin) were obtained from Sigma-Aldrich (Germany). Quercetin 3-*O*-glucoside, kaempferol 3-*O*-glucoside, isorhamnetin 3-*O*-glucoside and isorhamnetin 3-*O*-rutinoside were purchased from the National Standard Substances Center (Beijing, China). Acetonitrile, methanol, methyl *tert*-butyl ether (MTBE), all HPLC grade, were purchased from Alltech Scientific (Beijing, China).Acetone, n-hexane, butylated hydroxytoluene (BHT), HCl, NaCl and KOH were purchased from Sangon Company (Shanghai, China). HPLC-grade water was obtained from a Milli-Q System(Millipore, Billerica, MA, USA).

### Plant materials

The fifteen narcissus cultivars were grown in the horticultural farm of Shanghai Jiao Tong University, Shanghai, China. These cultivars were planted and grown under identical conditions, including fertilization, irrigation, and disease prevention methods. All the perianths and coronas were collected at the full-bloom stage, and stored at −80°C until use.

### Extraction of carotenoids

At the time of use, the frozen sample was ground to a fine powder in liquid nitrogen. 0.5g of powder was extracted with 15mL carotenoid extraction solution (n-hexane: acetone: absolute ethanol = 50:25:25, containing 0.01% BHT), shaken in a QL-861 vortex (Kylinbell Lab Instruments, Jiangsu, China), sonicated in a KQ-500DE ultrasonic cleaner (Ultrasonic Instruments, Jiangsu, China) for 30min at ambient temperature, then centrifuged in a SIGMA 3K15centrifuge (Sigma centrifuges, Germany) (4000rpm, 10min). The supernatant was collected and the extraction procedure was repeated three times. The supernatants from the sequential extractions were combined and washed with 10% aqueous NaCl. The organic layer was concentrated to dryness. The residue was dissolved in 2mL MTBE (containing 0.01% BHT) and saponified with 2mL 10% KOH–MeOH for 2 h at room temperature. Then the unsaponifiable matter was extracted with MTBE and washed with10% aqueous NaCl. The organic layer was dried over Na_2_SO_4_ and concentrated to dryness. The residue was dissolved in 1ml MTBE (containing 0.01% BHT), and spunin a SIGMA 3K15 centrifuge (12000rpm, 10min) before HPLC analysis. To avoid degradation and isomerization of carotenoids, extraction was performed in dim light using amber glassware. Three replicates were performed for each sample.

### Extraction of flavonoids

Exactly 0.5g of frozen sample was powdered in liquid nitrogen with a mortar and pestle, and extracted once using 5mL flavonoid extraction solution (methanol: water = 70:30, v/v, containing 0.1% HCl) vortexed for 1min, then sonicated in a KQ-500DE ultrasonic cleaner (Ultrasonic Instruments, Jiangsu, China) at 25°C for 30min.The supernatant was collected after centrifugation (SIGMA 3K15,4000rpm, 5min).The extraction process was repeated using 3mL and 2mL extraction solution respectively. All the extracts were combined and spunin a SIGMA 3K15centrifuge at 12000rpm for 10min before UPLC analysis. Three replicates were performed for each sample.

### HPLC-APCI-MS/MS analysis of carotenoid compounds

HPLC-MS analysis of carotenoids was carried out using an Agilent HPLC (Agilent, Waldbronn, Germany) equipped with a binary gradient pump (G1312B), a degasser unit (G1322A), a diode array detector (DAD) (G1315D), a thermo-autosampler (G1329B) and a column oven (G1316A). The HPLC system was coupled online to a Bruker Model Esquire 3000+ ion trap mass spectrometer (Bremen, Germany) fitted with an atmospheric pressure chemical ionization (APCI) source. The UV-visible spectra were obtained between 200 and 800nm, and the chromatograms were processed at 450nm. The MS parameters were set as follows: positive mode; current corona, 3500nA; source temperature, 350°C; dry gas, N_2_; temperature, 180°C; flow, 3.0L/min; nebulizer, 50 psi; MS/MS fragmentation energy, 1.4V. The mass spectra were acquired with a scan range of *m/z* from 100 to 800. The carotenoid separation was performed on a C_30_ YMC column (5μm, 250mm × 4.6mm) (YMC, Kyoto, Japan) using as mobile phase a linear gradient of A (acetonitrile: methanol = 75:25)/B (MTBE) from 95:5 to 50:50 over 30min, followed by 95:5 for 20min with a flow rate of 1 mL/min, and the column temperature set at 35°C.

The carotenoids were identified according to the following minimum criteria: accurate elution order on the C_30_ YMC column, the UV-visible spectrum (maximum absorption wavelengths (λ_max_), spectral fine structure (%III/II) and peak *cis* intensity (%A_B_/A_Ⅱ_) should be in agreement with the chromophore suggested, chromatographic properties should be verified in two systems or co-chromatography with authentic standards carried out, a mass spectrum should be obtained, allowing at least the confirmation of the molecular mass, and data should be consistent with available data from the literature [17,18].

### UPLC-Q-TOF-MS/MS analysis of flavonoid compounds

Chromatographic analysis of flavonoids was performed on an ACQUITY UPLC system (Waters, Milford, USA) equipped with a binary solvent delivery manager, a sample manager and a diode-array detector (DAD). A reverse phase column ACQUITY UPLC BEH C18 (1.7 μm, 100 mm × 2.1 mm) was used and eluted with a linear gradient of A (0.1% aqueous formic acid solution) and B (0.1% formic acid in acetonitrile) at a flow rate of 0.4mL/min and a temperature of 45°C. The elution program was set as follows: initial, 95% A, 5% B; 0.5 min, 95% A, 5% B; 7 min, 80% A, 20% B; 13 min, 70% A, 30% B; 13.5 min, 50% A, 50% B; 14.5 min, 15% A, 85% B; 15 min, 0% A, 100%B; 17.5min, 0% A,100% B; 18min, 95% A, 5% B; 20min, 95%A, 5% B. The injection volume was 2μL. The spectra data was recorded from 100 to 1000nm, and the chromatograms were processed at 350nm.

MS data were recorded using a Waters Micromass Q-TOF Premier Mass Spectrometer equipped with an electrospray interface (Waters, Milford, USA).The mass spectra were collected in the 50–1000*m/z* range within negative- and positive-ion modes. For analysis, the electrospray source parameters were as follows: capillary voltage, 2.6kV; sampling cone, 55V; collision energy, 4eV; source temperature, 100°C; desolvation temperature, 300°C; desolvation gas, 500 L/hr. The accurate mass and composition for the precursor and fragment ions were calculated using MassLynx 4.1 software.

### Preparation of standards

The all-*trans*-*β*-carotene and rutin standards were accurately weighed and dissolved in MTBE (containing 0.01% BHT) and methanol, respectively. Then they were diluted to appropriate concentrations, 0.05–40μg/mL for all-*trans*-*β*-carotene and 6.25–800μg/mL for rutin. For the analytical curves of carotenoids and flavonoids, the *r* ^2^ were 0.999 and 0.998, the limits of detection (LOD) were 0.017 and 0.59μg/mL, and the limits of quantification (LOQ) were 0.056 and 2.07μg/mL respectively.

### Perianth and corona color measurement

**Fig 2:**
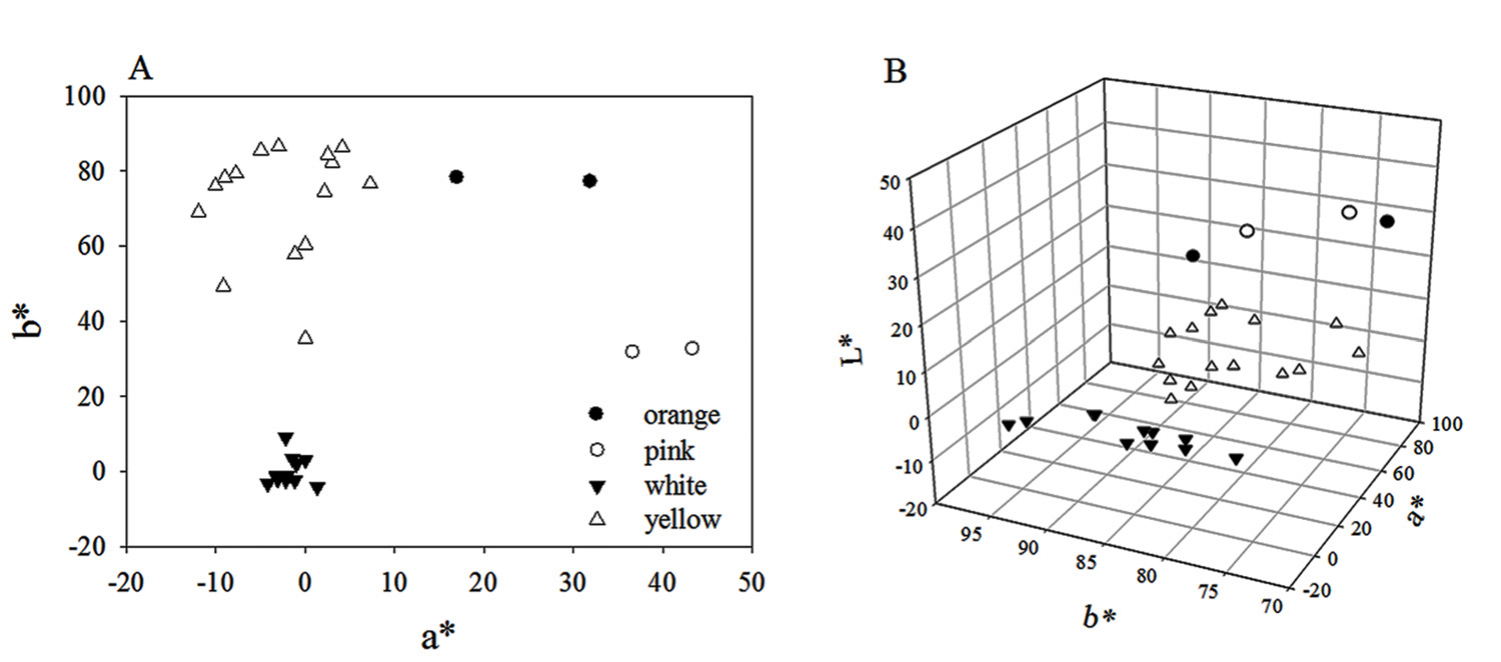
Flower color distribution of narcissus cultivars in coordinate systems of bivariate (a* and b*) (A) and trivariate (a*, b*, and L*) (B), respectively. The flower colors were identified by the RHSCC value.

The Royal Horticultural Society Color Chart (RHSCC) and an NF333 spectrophotometer (Nippon Denshoku Industries Co. Ltd., Japan) were used for recording the color parameters of fresh perianth and corona (Table 1). The differences of sample chromaticity were measured by CIE 1976 L*a*b*(CIELAB), which contained L*, a*, and b*parameters to describe all aspects of the color. In the CIE 1976 L*a*b* system, the L* value represents lightness of shade ranging from 0 (black) to 100 (white), the color parameters a* and b*positive are for red and yellow, negative are for green and blue. In addition, chroma C* and hue angle *h* value correspond to the saturation and hue of the color [19].They are calculated from parameters a*and b* [C* = (a*^2^+b*^2^)^1/2^] [*h* = arctan b*/a*]. The color coordinates measured are shown in Fig 2: the L*values ranged from 71.37 to 95.48, a* values from -11.96 to 43.37 and b* values from -4.19 to 86.52.

**Table 1:** Perianths and coronas colors and color parameters of fifteen narcissus cultivars.

sample^*a*^		colour	RHSCC^*b*^	CIEL*a*b*^c^					TF^*d*^mg/g	TC^*e*^mg/g	CI^*f*^
				L*	a*	b*	C*	*h*			
avalon	perianth	light yellow	4C	87.69	-9.18	49.20	50.05	-1.39	9.04	0.05	180.80
	corona	yellow	5C	92.10	-10.07	75.99	76.65	-1.44	5.63	0.07	80.43
decoy	perianth	milk white	155B	95.48	-4.21	-3.25	5.32	0.65	13.80	0.00	∞
	corona	pink	38B	71.37	43.37	32.61	54.26	0.64	7.74	0.77	10.06
gigantic star mutation	perianth	white	157D	83.21	-2.19	-2.30	3.17	0.80	9.19	0.01	919
	corona	light yellow	3B	83.01	-1.17	57.87	57.88	-1.55	5.36	0.04	134
jack snipe	perianth	white	NN155C	94.13	-3.26	-1.31	3.51	0.38	15.37	0.03	512.33
	corona	yellow	9A	92.11	-4.98	85.56	85.70	-1.51	7.91	0.90	8.79
lemon beauty	perianth	white	NN155C	80.48	-2.01	-1.07	2.27	0.48	10.13	0.01	1013
	corona	yellow white	6A	77.45	0.00	60.31	60.31	∞	9.03	0.17	53.12
marieke	perianth	yellow	9A	80.79	-7.79	79.37	79.75	-1.47	10.46	0.41	25.51
	corona	dark yellow	14A	87.25	4.13	86.26	86.35	1.52	7.32	0.91	8.04
mondragon	perianth	milk white	155B	88.62	0.00	3.08	3.08	∞	4.97	0.03	165.67
	corona	light orange	17B	89.07	17.03	78.22	80.52	1.36	5.04	0.37	13.62
mount hood	perianth	white	157C	80.09	1.32	-4.19	4.39	-1.26	6.92	0.00	∞
	corona	light yellow	3C	86.11	0.00	35.34	35.34	∞	5.15	0.01	515
pink charm	perianth	white	NN155C	84.32	-0.99	2.06	2.29	-1.12	11.75	0.00	∞
	corona	pink white	29C	79.25	36.68	31.77	48.52	0.71	7.68	0.06	128
pinza mutation	perianth	yellow	5B	73.65	2.14	74.40	74.43	1.54	10.02	0.50	20.04
	corona	light orange	23B	72.10	31.90	77.15	83.48	1.18	5.15	0.95	5.42
shangnong dieying	perianth	milk white	155B	85.27	-3.11	-2.09	3.74	0.59	9.84	0.04	246
	corona	yellow	9A	83.75	2.99	82.22	82.27	1.53	5.44	0.72	7.55
shangnong ruhuang	perianth	white	157C	76.21	-1.18	-2.37	2.64	1.11	11.73	0.03	391
	corona	yellow	6C	75.90	7.26	76.58	76.92	1.48	4.22	0.47	8.98
slim whitman	perianth	white	157C	83.77	-1.47	3.44	3.74	-1.17	7.6	0.02	380
	corona	yellow	6A	88.07	2.51	84.32	84.02	1.54	3.59	0.18	19.94
spellbinder	perianth	yellow	3A	88.16	-11.96	68.98	70.01	-1.40	13.2	0.21	62.86
	corona	yellow	4B	87.27	-9.01	78.25	78.77	-1.46	7.77	0.10	77.70
valdrome	perianth	white	157C	89.61	-2.21	9.08	9.34	-1.33	7.55	0.02	377.5
	corona	yellow	3A	90.09	-3	86.52	86.57	-1.54	5.36	0.43	12.47

^*a*^ The name of narcissus cultivars.

^*b*^ RHSCC: Royal Horticultural Society Color Chart.

^*c*^ L*, lightness; a*, b*, chromatic components; C*, chroma; C* = (a*^2^+ b*^2^)^1/2^, *h*, hue angle (u), *h* = arctan(b*/a*).

^*d*^TF: total flavonoids in mg per 1 g fresh samples.

^*e*^TC: total carotenoids in mg per 1 g fresh samples.

^*f*^CI: copigment index = TF/TC; ‘∞’: means samples without carotenoids.

## Results and Discussion

### Identification of carotenoid compounds

**Fig 3:**
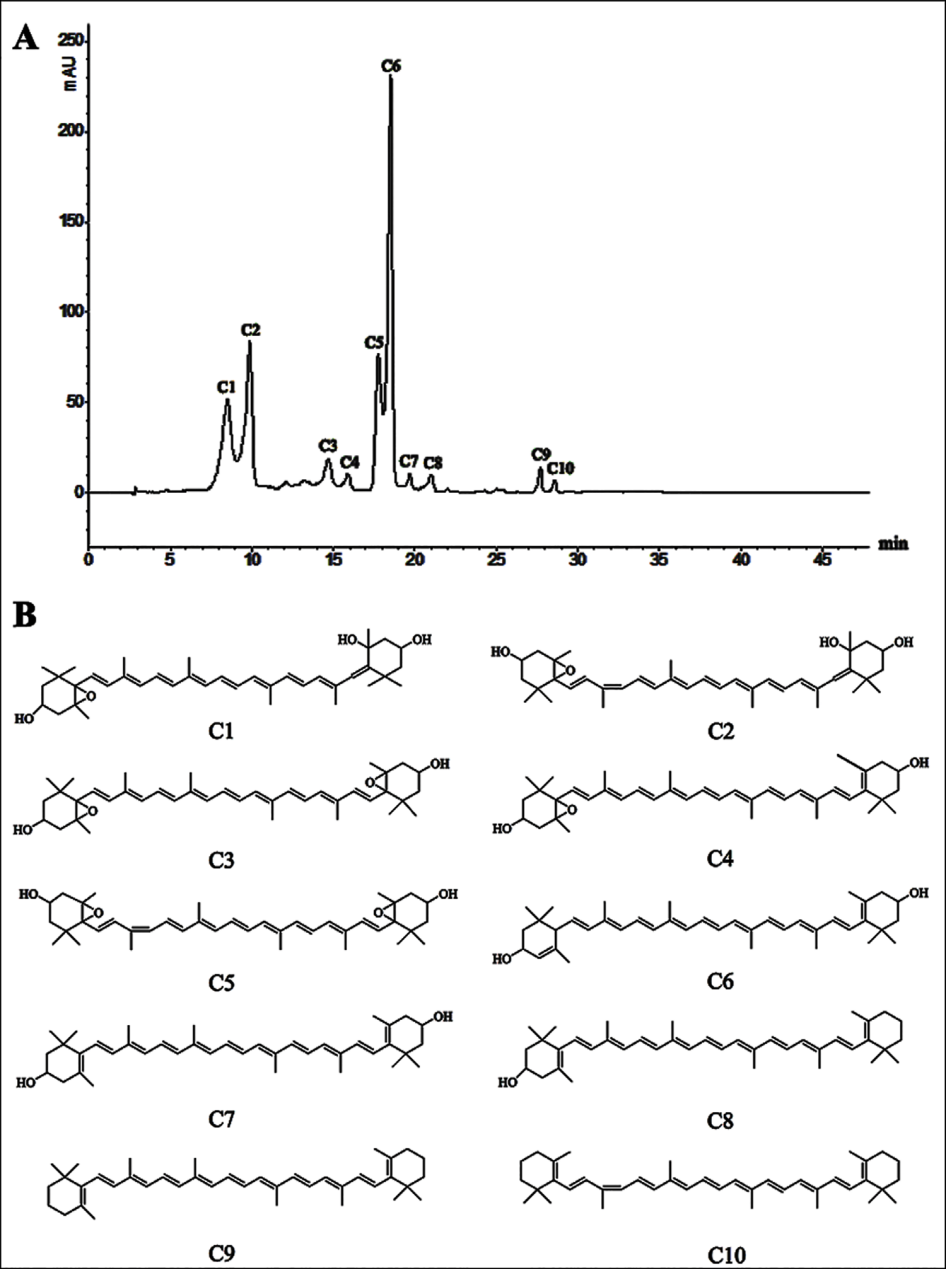
The HPLC chromatogram (A) and chemical structure scheme (B) of carotenoids from narcissus.

The HPLC-DAD-APCI-MS/MS method allowed the separation and tentative identification of 10 carotenoids in narcissus. A typical chromatogram and chemical structures of the 10 carotenoids are shown in Fig 3, and their characteristics are presented in Table 2. The mean content (mg/g) of carotenoid compounds in each narcissus cultivar was shown in S1 Table.

**Table 2:** Carotenoids compounds detected in narcissus cultivars by HPLC-APCI-MS/MS.

peak^*a*^	tentative compound	tR^*b*^(min)	λmax (nm)	%III/II	%A_B_/A_II_	[M+H]^+^ (m/z)	MS/MS fragment ions(+)(m/z)	ID^*d*^
C1	all-*trans*-neoxanthin	8.48	417,441,470	n.c.^*c*^	n.c.	601	583[M+H-18]+, 565[M+H-18-18]+, 547[M+H-18-18-18] +, 509[M+H-92] +, 491, 393	std
C2	9-*cis*-neoxanthin	9.88	327,416,438,469	76	8	601	583[M+H-18] ^+^, 565[M+H-18-18] ^+^, 547[M+H-18-18-18] ^+^, 509[M+H-92], 393	ref 17,18,20,21
C3	all-*trans*-violaxanthin	14.67	415,439,468	n.c.	n.c.	601	583[M+H-18] ^+^, 565[M+H-18-18] ^+^, 509[M+H-92] ^+^, 491[M+H-92-18], 221	std
C4	all-*trans*-antheraxanthin	15.91	420,444,474	59	0	585	567[M+H-18] ^+^, 549[M+H-18-18] ^+^, 529[M+H-56] ^+^, 221	ref 18,24
C5	9-*cis*- violaxanthin	17.78	326,413,436.466	75	11	601	583[M+H-18] ^+^, 565[M+H-18-18] ^+^, 509[M+H-92] ^+^, 221	ref 18,21
C6	all-*trans*-lutein	18.56	420,444,472	n.c.	n.c.	569	551[M+H-18] ^+^, 533[M+H-18-18] ^+^, 495[M+H-18-56]^+^, 477[M+H-92] ^+^	std
C7	all-*trans*-zeaxanthin	20.76	423,450,476	45	0	569	551[M+H-18] ^+^, 533[M+H-18-18] ^+^	ref 18,22,24
C8	all-*trans*-β-cryptoxanthin	21.12	421,448,475	n.c.	n.c.	553	535[M+H-18] ^+^, 495, 461[M+H-92] ^+^	std
C9	all-*trans*-β-carotene	27.80	421,450,478	n.c.	n.c.	537	444[M-92] ^+^	std
C10	9-*cis*-β-carotene	29.76	340,420,447,475	27	19	537	444[M-92] ^+^	ref 17,21,22

^*a*^Numbered according to Fig 3.

^*b*^tR: retention time.

^*c*^n.c.: not calculated.

^*d*^ID: std, standard compound; ref, reference.

Compound C1 was protonated at *m/z* 601 and produced a class of fragments at *m/z* 583[M+H-18]^+^,565[M+H-18-18]^+^ and 547[M+H-18-18-18]^+^ in subsequent MS/MS experiments, due to consecutive losses of three hydroxyl groups. Considering the elution time, UV-visible and mass spectra features compared with an authentic standard and previous studies [17,18,20–22], compound C1 was assigned as all-*trans*-neoxanthin. It was observed that compound C2 eluted after all-*trans*-neoxanthin and had the same protonated molecule and similar fragments. Moreover, C2 showed a hypsochromic shift of 3nm, decreased spectral fine structure and high intensity of the *cis*-peak for the *cis*-isomer. The appearance of an additional “*cis*-peak” with low intensity at 327nm proved the presence of a peripheral double bond [22,23].Therefore, compound C2 was identified as 9-*cis*-neoxanthin.

By comparison of the elution order, λ_max_ values, UV-visible and mass spectrometric characteristics with the literature [18,24], compound C4 was assigned as all-*trans*-antheraxanthin. As expected, the protonated molecule was observed at *m/z* 585, with fragments at *m/z* 567[M+H-18]^+^ and 549[M+H-18-18]^+^ resulting from the consecutive losses of two hydroxyl groups. In addition, the fragment at *m/z* 221 revealed that the epoxide was in a ring with a hydroxyl group.

Taking into consideration its elution behavior, UV-visible spectroscopy features, the presence of a protonated molecule at *m/z* 601 and characteristic dehydrated fragments at *m/z* 583[M+H-18]^+^, 565[M+H-18-18]^+^ and 221, compound C3 was identified as all-*trans*-violaxanthin. Compound C5 (9-*cis*-violaxanthin) eluted after all-*trans*-violaxanthin, and had a similar fragmentation pathway in MS^2^ experiments. This was corroborated by observation of lower λ_max_ values, spectral fine structure and high *cis*-peak intensity, and by the appearance of an additional “*cis*-peak” with low intensity at 326nm, indicating the presence of a peripheral double bond.

All-*trans*-lutein (compound C6) was identified through UV-visible and mass spectra characteristics in comparison with an authentic standard and with the previous literature [17,18,21,22]. All-*trans*-zeaxanthin (compound C7) had the same chemical formula (C_40_H_56_O_2_), protonated molecule (*m/z* 569), and characteristic dehydrated fragments at *m/z* 551[M+H-18]^+^ and 533[M+H-18-18]^+^as all-*trans*-lutein. Therefore, they were distinguished according to the UV-visible and mass spectra. The λ_max_ values of zeaxanthin were 423, 450, 476nm, which were higher than those of lutein (420, 444, 472nm).In addition, MS indicated a more intense protonated molecule peak (*m/z* 569) compared to the fragment at *m/z* 551, with lutein showing the opposite result. In addition, previous data demonstrated the MS^2^ fragments at *m/z* 495 to be indicative of lutein [18,22,24].

According to the λ_max_ values, fine structure and MS features given in the literature [17,20–22],compound C8 was characterized as all-*trans*-*β*-cryptoxanthin. This was confirmed by the identification of a protonated molecule at *m/z* 553, and the fragments at *m/z* 535[M+H-18]^+^ and 461[M+H-92]^+,^ resulting from the elimination of a hydroxyl group and toluene, respectively. Moreover, compound C8 exhibited identical retention behavior as the authentic standard.

Elution order depended on the number of hydroxyl groups in the rings [21]. The more hydroxyl groups that the carotenoids had, the faster they eluted. On the basis of the characteristics above, corresponding standards, UV-visible and mass spectra, C9 was identified as all-*trans*-*β*-carotene. Compared to compound C9, C10 showed the same protonated molecule at *m/z* 537 and the fragment at *m/z* 444[M-92], due to the loss of toluene, but revealed a hypsochromic shift of 3nm and an additional “*cis*-peak” at 340nm. Therefore, it was identified as 9-*cis*-*β*-carotene.

Among the 10 carotenoids identified in narcissus in this study, all-*trans*-lutein, all-*trans*-*β*-cryptoxanthin, all-*trans*-*β*-carotene and all-*trans*-zeaxanthin have been previously reported by other researchers [14,17]. This is the first report of the other six carotenoids in narcissus. It is worth mentioning that the MS technique was not applied in any of the previous reports, which used column chromatography to separate and identify carotenoids in *Narcissus pseudonarcissus* ‘King Alfred’ and *Narcissus poeticus* ‘Scarlet Elegance’ [14,17].

### Identification of flavonoid compounds

**Fig 4:**
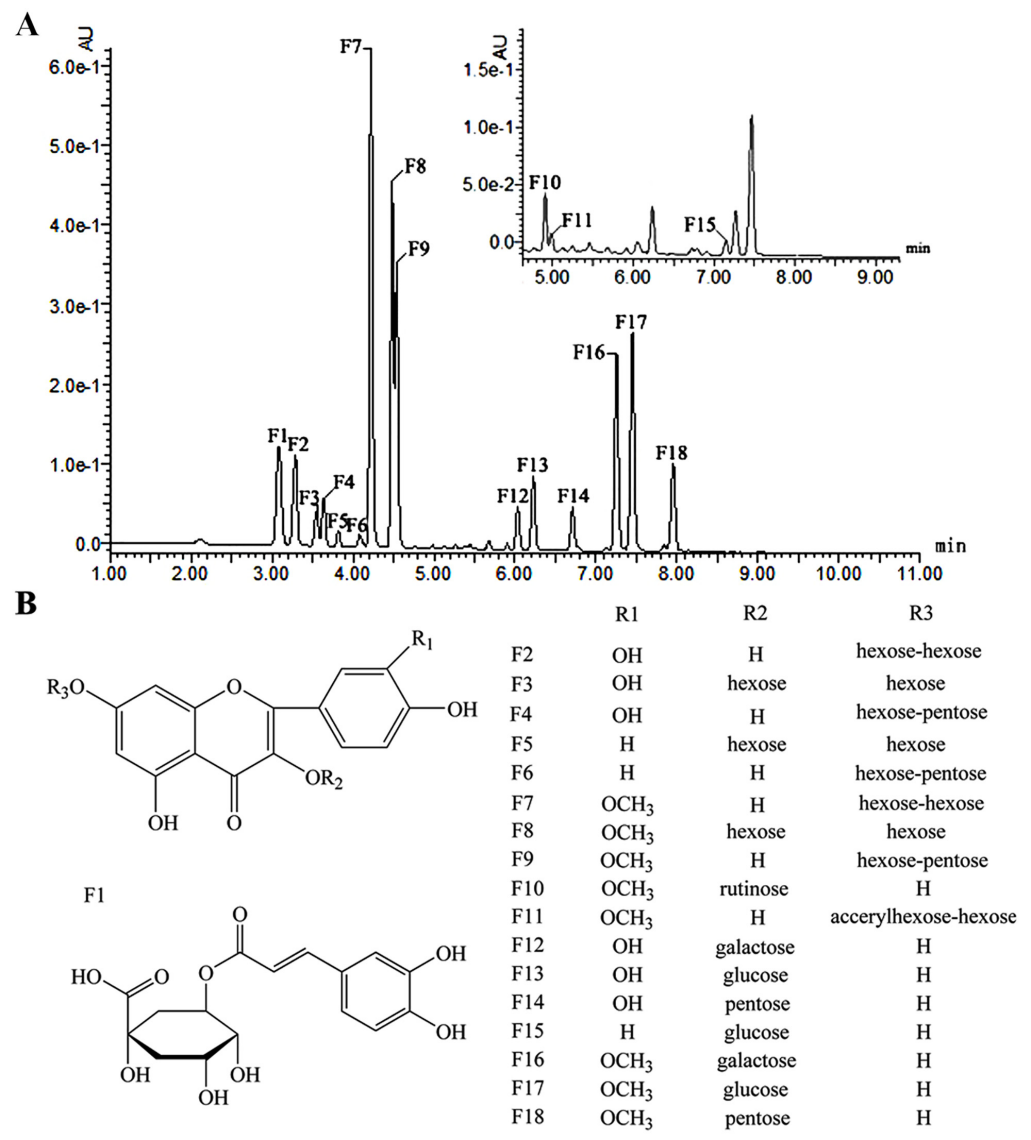
The UPLC chromatogram (A) and chemical structure scheme (B) of flavonoids from narcissus.

Using analysis by UPLC-Q-TOF-MS/MS, we identified, or tentatively identified, seventeen flavonol glycosides and chlorogenic acid. Three aglycones of flavonol were characterized as quercetin (F2, F3, F4, F12, F13 and F14), kaempferol (F5, F6 and F15) and isorhamnetin (F7, F8, F9, F16, F17 and F18) through UV-visible and mass spectra features in comparison with the previous literature [25,26]. Typical chromatograms and chemical structures are shown in Fig 4. It was observed, as expected, that the separation of the eighteen compounds followed the reverse-phase pattern of flavonoid *O*-glycosides >flavonoid *O*-diglycosides >cinnamic acid (Table 3) [27]. The mean content (mg/g) of flavonoid compounds in perianths and coronas of fifteen narcissus cultivars were shown in S2 Table.

**Table 3:** Flavonoids compounds detected in narcissus cultivars by UPLC-Q-TOF-MS/MS.

peak^*a*^	tentative compound	tR^*b*^(min)	λmax (nm)	ESI-PI MS/MS (m/z)	ESI-NI MS/MS (m/z)	ID^*c*^
F1	chlorogenic acid	3.10	237,245,330	355[M+H]^+^(45), 193 [M+H-162](100)	353[M-H]^-^(40), 191 [M-H-162](100)	ref 24,33
F2	quercetin 7-*O*-dihexoside	3.30	256,365	627[M+H]^+^(65), 465[M+H-162](89), 303[Y_0_^+^](100)	625[M-H]^-^(60), 463[M-H-162](20), 301[Y_0_^-^](45),	ref 26,29,30
F3	quercetin 3,7-di-*O*-hexoside	3.56	265,355	627[M+H]^+^(45), 465[M+H-162](65), 303[Y_0_^+^](100)	625[M-H]^-^(40), 463[M-H-162](100), 301[Y_0_^-^](35)	ref 25,31,34
F4	quercetin 7-*O*-hexoside-pentoside	3.65	256,365	597[M+H]^+^(55), 465[M+H-132](50), 303[Y_0_^+^](100)	595 [M-H]^-^(55), 463[M-H-132](15), 301[Y_0_^-^](30),	ref 36
F5	kaempferol 3,7-di-*O*-hexoside	3.83	266,346	611[M+H]^+^(64), 449[M+H-162](100), 287[Y_0_^+^](55)	609[M-H]^-^(45), 447[M-H-162](100), 285[Y_0_^-^](26),	ref 25,30,31
F6	kaempferol 7-*O*-hexoside-pentoside	4.08	265,358	581[M+H]^+^(65), 449[M+H-132](45), 287[Y_0_^+^](100)	579[M-H]^-^(40), 447[M-H-132](15), 285[Y_0_^-^](38)	ref 26,36
F7	isorhamnetin 7-*O*-dihexoside	4.23	254,365	641[M+H]^+^(85), 479[M+H-162](100), 317[Y_0_^+^](95)	639[M-H]^-^(100), 477[M-H-162](35), 315[Y_0_^-^](30),	ref 29,31,32
F8	isorhamnetin 3,7-di-*O*-hexoside	4.49	255,353	641[M+H]^+^(88), 479[M+H-162](70), 317[Y_0_^+^](100)	639[M-H]^-^(85), 477[M-H-162](100), 315[Y_0_^-^](20)	ref 25,31
F9	isorhamnetin 7-*O*-hexoside-pentoside	4.55	255,365	611[M+H]^+^(70), 479[M+H-132](50), 317[Y_0_^+^](100)	609[M-H]^-^(50), 477[M-H-132](15), 315[Y_0_^-^](35)	ref 36
F10	isorhamnetin 3-*O*-rutinoside	4.91	254,354	625[M+H]^+^(10), 479[M+H-146](100),317[Y_0_^+^,M+H-308](45)	623[M-H]^-^(30), 477[M-H-146](40), 315[Y_0_^-^, M-H-308](80)	std
F11	isorhamnetin 7-*O*-accerylhexoside-hexoside	5.11	254,365	706[M+Na]^+^(23), 683[M+H]^+^(55), 521[M+H-162]^+^(16), 317[Y_0_^+^](100)	681[M-H]^-^(90), 519[M-H-162](40), 315[Y_0_^-^](30), 314[Y_0_^—^H]^.-^ (10)	ref 36
F12	quercetin 3-*O*-galactoside	6.05	255,352	465[M+H]^+^(10), 303[Y_0_^+^,M+H-162] (100)	463[M-H]^-^(70), 301[Y_0_^-^,M-H-162](20), 300[Y_0_-H]^.-^(35)	ref 26–28
F13	quercetin 3-*O*-glucoside	6.24	257,354	487[M+Na]^+^(10), 465[M+H]^+^(20), 303[Y_0_^+^,M+H-162](100)	463[M-H]^-^(45), 301[Y_0_^-^,M-H-162](15), 300[Y_0_-H]^.-^(25)	std
F14	quercetin 3-*O*-pentoside	6.73	256,354	435[M+H]^+^(15), 303[Y_0_^+^](100)	433[M-H]^-^(50), 301[Y_0_^-^](35)	ref 27,28
F15	kaempferol 3-*O*-glucoside	7.15	267,351	449[M+H]^+^(15), 287[Y_0_^+^](100)	447[M-H]^-^(65), 285[Y_0_^-^](10), 284[Y_0_-H]^.-^(28)	std
F16	isorhamnetin 3-*O*-galactoside	7.27	254,354	479[M+H]^+^(15), 317[Y_0_^+^](100)	477[M-H]^-^(100), 315[Y_0_^-^](15), 314[Y_0_-H]^.-^(30)	ref 26–28
F17	isorhamnetin 3-*O*- glucoside	7.46	255,354	479[M+H]^+^(20), 317[Y_0_^+^](100)	477[M-H]^-^(100), 315[Y_0_^-^](20), 314[Y_0_-H]^.-^(35)	std
F18	isorhamnetin 3-*O*- pentoside	7.97	254,353	471[M+Na]^+^(15), 449[M+H]^+^(10), 317[Y_0_^+^](100)	447[M-H]^-^(85), 315[Y_0_^-^](20), 314[Y_0_-H]^.-^(45)	ref 27,28

^*a*^Numbered according to Fig 4.

^*b*^tR: retention time.

^*c*^ID: std, standard compound; ref, reference.

In general, the glycosylation of flavonoids occurs at the 3- and 7-position of aglycone in flavonol. The level of the relative abundance of the aglycone ion [Y_0_^-^] and aglycone ion free radical [Y_0_^—^H]^.-^ determines the location of the glycosylation as described in previous studies [25,26]. If a glycoside is located at the 3-position of aglycone, the relative abundance of [Y_0_^—^H]^.-^ is higher than that of [Y_0_^-^], and the converse occurs when glycosylation occurs at the 7-position. Considering the conclusion above, mass spectra characteristics and the retention time compared with the standards, compounds F10, F13, F15 and F17 were identified as isorhamnetin 3-*O*-rutinoside, quercetin 3-*O*-glucoside, kaempferol 3-*O*-glucoside and isorhamnetin 3-*O*-glucoside. Meanwhile, compounds F14 and F18 were assigned as quercetin 3-*O*-pentoside and isorhamnetin 3-*O*-pentoside based on the similar behavior of the fragments compared with F13 and F17 [27,28]. It is worth noting that compounds F12 and F16 shared identical molecular ions, aglycone ions and fragment ions with F13 and F17, except for very small differences in their relative abundance and UV characteristic absorption wavelength. Given their different elution behavior and previously reported data [26–28], compounds F12 and F16 were tentatively assigned as quercetin 3-*O*-galactoside and isorhamnetin 3-*O*-galactoside.

On the basis of previous studies [29–31], a typical flavonol UV spectrum consisted of two *λ*_max_ peaks at 250–295 nm (band II) and 310–370 nm (band I), and substituents in different positions could alter the wavelength and relative intensities of these maxima. The UV spectra of kaempferol 3-*O*-glycosides and 3,7-*O*-diglycosides showed a *λ*_max_ around 266 and 348 nm, and quercetin or isorhamnetin 3-*O*-glycosides and 3,7-*O*-diglycosidesshowed a *λ*_max_ around 256 and 354 nm respectively. By contrast, the characteristic UV *λ*_max_ of kaempferol 3-*O*-glycosides and 7-*O*-glycosides was approximately 266 and 366 nm respectively, whereas 7-*O*-glycosides in quercetin or isorhamnetin had a UV *λ*_max_ around 256 and 370 nm respectively.

The MS fragmentation data of compound F2, *m/z* 627[M+H]^+^, 465[M+H-162] and 303[Y_0_^+^] in PI mode and *m/z* 625[M-H]^-^, 463[M-H-162] and 301[Y_0_^-^] in NI mode, suggested two hexose rings were the glycosyl substituent. Considering the UV characteristic absorption wavelength (256,365 nm), the relative abundance of [Y_0_^—^H]^.-^ and [Y_0_^-^], MS data and the previous literature[29,32,33], compound F2 was finally deduced as quercetin 7-*O*-dihexoside.In the same way, compound F7 was tentatively characterized as isorhamnetin 7-*O*-dihexoside [29,31,32]. Compound F3 had the same molecular ion, aglycone ion and fragment ions with F2, apart from the UV *λ*_max_ (265,355 nm) and relative abundance, especially the relative abundance of 463[M-H-162], was much higher than that of F2. By comparison with previous reports [25,31,34], compound F3 was deduced as quercetin 3,7-di-*O*-hexoside. The fragmentation behaviors of compounds F5 and F8 were similar to quercetin 3,7-di-*O*-hexoside in MS/MS analysis, and **t**he UV spectra of which showed *λ*_max_ at 266,346 nm and 255,353 nm, therefore, they were assigned as kaempferol 3,7-di-*O*-hexoside and isorhamnetin 3,7-di-*O*-hexoside, respectively [25,30,31].

Based on the MS data, compound F4 was determined to have two substituents; one hexose and one pentose molecule. The MS/MS fragment *m/z* 463[M-H-132]^-^ was present without a *m/z* 433[M-H-162]^-^ peak. This suggested that quercetin was glycosylated by a disaccharide. The relative abundance of the aglycone ion *m/z* 303[Y_0_^+^] was higher than that of the fragment ion *m/z* 465[M+H-132]. This suggested that the glycosidic bond of the disaccharide was 1→2 connection. In addition, according to previous conclusions [26,35], the [Y_0_^—^H]^.-^ ion is prone to be generated in the case of a 1, 2-linkage between the two monosaccharide residues in disaccharide derivatives. As an exception, the aglycone ion free radical 300[Y_0_^—^H]^.-^ was observed in MS/MS data. Taken together, compound F4 was therefore tentatively characterized as quercetin 7-*O*-hexoside-pentoside. In the same way, compounds F6, F9 and F11 were tentatively characterized as kaempferol 7-*O*-hexoside-pentoside, isorhamnetin 7-*O*-hexoside-pentoside and isorhamnetin7-*O*-acerylhexoside-hexoside, respectively.

One hydroxycinnamic acid derivative was detected based on the characterization of UV–visible absorption spectroscopy readings at 237, 245 and 330nm, which were similar to the typical absorption peaks of chlorogenic acid. Compound F1 revealed the presence of a protonated molecule at *m/z* 353 and the ion fragment corresponding to the deprotonated quinic acid at *m/z* 191. Therefore, F1 was identified as chlorogenic acid [27,36].

It was challenging to identify the flavonoids in narcissus due to the lack of previous literature in this area. This is the first time that the composition of flavonoids in narcissus has been characterized, and it was clear that flavonols constitute the major group of flavonoid compounds. It is well known that flavonoids are important antioxidants, among which the flavonols quercetin and kaempferol have the highest antioxidant activity [25].As shown in Table 1, it is clear that the total content of flavonoids (TF) was high in narcissus. Therefore we predict that the abundance of flavonoids in narcissus will be a good resource for bioactivity for future utilization.

### Relationship between color parameters and pigment composition

We then analyzed pigment color and its relationship with carotenoid and flavonoid levels across fifteen different narcissus cultivars. Pigment composition is the most critical aspect of flower color [3,5].Multiple linear regression (MLR) analysis was used to explore the relationship between color formation and pigment composition [6,37]. L*, a*, b*, C*and *h* were chosen as dependent variables, 31 contents of pigment components (including ten carotenoids, seventeen flavonols and chlorogenic acid, TC (total carotenoid content), TF (total flavonoid content) and CI (co-pigment index = TF/TC) were independent variables. Significant statistical results were as follows (p<0.05):

L* = 77.904+1.398 all-*trans*-violaxanthin-9.401all-*trans*-*β*-cryptoxanthin (R^2^ = 0.478)

a* = 45.263–5.528 all-*trans*-violaxanthin-2.979 9-*cis*-*β*-carotene-1.691TF+0.02CI (R^2^ = 0.927)

b* = 39.214–0.074CI+6.684all-*trans*-violaxanthin+50.014TC-5.819quercetin7-O-dihexoside(R^2^ = 0.914)

C* = 53.750–0.061CI-1.9479-*cis*-*β*-carotene+44.181TC+4.403all-*trans*-violaxanthin-Quercetin 7-*O*-dihexoside (R^2^ = 0.891)

*h* = 0.445–0.258all-*trans*-violaxanthin+0.322 quercetin 7-*O*-dihexoside (R^2^ = 0.462)

The MLR analysis showed that all-*trans*-violaxanthin was the major factor that affected color parameters, with positive effects on the value of L*, b* and C*, but negative effects on the values of a* and *h*. The secondary factors quercetin7-*O*-dihexoside and CI had negative effects on the values of b* and C*, and positive effects on the values of *h* and a*, respectively. TC played a positive role in the value of b*and C*, and 9-*cis*-*β*-carotene played a negative role in the value of a* and C*. In addition, CI played a positive role in the value of a*, and all-*trans*-*β*-cryptoxanthin played a negative role in the value of L*.

MLR analysis showed that there are many factors affecting narcissus flower color:all-*trans*-violaxanthin, all-*trans*-*β*-cryptoxanthin, 9-*cis*-*β*-carotene, quercetin 7-*O*-dihexoside, TC, TF and CI. Among these, all-*trans*-violaxanthin and TC are the main factors. We could see that increasing the contents of all-*trans*-violaxanthin and TC, the value of L*, b* and C* increased while a* decreased, which means that the flower color changed to become more yellow, green and much more vivid. From Fig 1, we know that most of the narcissus flowers are yellow, and the color parameters b*positive was for yellow. Taking into consideration the correlations between the color parameters b* and TC and TF, it was shown that although TF was higher than TC, it had no correlation with b* and conversely TC had positive effects on b*. Increasing TC, the value of b* increased, and the yellow color turned deeper. That is to say, the yellow color of narcissus flower maybe due to carotenoids levels. In addition, comparison of HPLC and MLR analysis, showed that all-*trans*-lutein was the major carotenoid in fifteen narcissus cultivars, however, it did contribute to the MLR analysis and conversely all-*trans*-violaxanthin replaced it. So we could speculate from the conclusion above that the shade of color might not depend on the size of relative peak area of the carotenoid compounds.

It is worth mentioning that the coronas of cultivars decoy and pink charm are white and orange pink, generally, pink color is derived from red anthocyanin or red-colored carotenoid lycopene. However, the flower pigments in narcissus perianth and corona were carotenoids and flavonls only, the anthocyanin was not existent, based on our previous studies. Using analysis by HPLC-DAD-MS/MS, we observed, lycopene didn’t exist in pink corona of cultivars, or it might be existent but under the detection line. Therefore, the orange pink coronas of cultivars decoy and pink charm attributed largely to the accumulation of β-carotene.

In summary, we have reported the results of a detailed investigation demonstrating that the perianths and coronas of narcissus have unique carotenoid and flavonoid compositions. Using powerful analytical tools, HPLC-APCI-MS/MS and UPLC-Q-TOF-MS/MS, ten carotenoids, seventeen flavonols and chlorogenic acid were identified, and six carotenoids, all flavonols and chlorogenic acid are reported for the first time in narcissus. With the carotenoid and flavonoid composition data, a flower pigment fingerprinting data base of narcissus could be established. Additionally, we also studied the relationship between flower color and pigment composition. This could obviously provide a complete understanding of flower color formation, and help to select appropriate parental strains for hybridization and breeding.

## Supporting Information

S1 Table Occurrence of carotenoid compounds in perianths and coronas of fifteen narcissus cultivars. (DOCX)

S2 Table Occurrence of flavonoid compounds in perianths and coronas of fifteen narcissus cultivars. (DOCX)
