# Supplementary material for: Composition of Carotenoids and Flavonoids in Narcissus Cultivars and their Relationship with Flower Color
Source: PLoS One. 2015 Nov 4;10(11):e0142074. doi: 10.1371/journal.pone.0142074 (PMC4633037; doi:10.1371/journal.pone.0142074)
Supplement: S1 Table — (DOCX) [file pone.0142074.s001.docx]

**S1 Table The mean content (mg/g) of carotenoid compounds in perianths and coronas of fifteen narcissus cultivars.**

| Sample | Part | C1*^a^* | C2 | C3 | C4 | C5 | C6 | C7 | C8 | C9 | C10 | TC(mg/g) |
| --- | --- | --- | --- | --- | --- | --- | --- | --- | --- | --- | --- | --- |
| avalon | perianth | 0.003 | 0.004 | 0.006 | 0.009 | 0.005 | 0.017 | —*^b^* | — | 0.005 | 0.001 | 0.05 |
|  | corona | 0.005 | 0.009 | 0.003 | 0.004 | 0.008 | 0.021 | — | — | 0.016 | 0.004 | 0.07 |
| decoy | perianth | — | — | — | — | — | — | — | — | — | — | 0.00 |
|  | corona | — | — | — | — | — | — | — | — | 0.68 | 0.09 | 0.77 |
| gigantic-star-mutation | perianth | — | — | — | — | 0.001 | 0.003 | — | — | 0.005 | 0.002 | 0.01 |
|  | corona | 0.005 | 0.012 | 0.008 | 0.003 | 0.004 | 0.05 | — | 0.003 | 0.008 | 0.002 | 0.04 |
| jack-snipe | perianth | 0.003 | 0.006 | — | — | — | — | — | — | 0.015 | 0.006 | 0.03 |
|  | corona | 0.12 | 0.31 | 0.09 | 0.02 | 0.03 | 0.23 | — | — | 0.06 | 0.01 | 0.90 |
| lemon-beauty | perianth | 0.002 | 0.003 | — | — | — | — | — | — | 0.005 | — | 0.01 |
|  | corona | 0.024 | 0.041 | 0.01 | 0.004 | 0.004 | 0.009 | — | — | 0.072 | 0.006 | 0.17 |
| marieke | perianth | 0.02 | 0.08 | 0.03 | 0.01 | 0.05 | 0.17 | — | 0.01 | 0.03 | 0.01 | 0.41 |
|  | corona | 0.07 | 0.18 | 0.05 | 0.01 | 0.08 | 0.45 | 0.01 | 0.01 | 0.04 | 0.01 | 0.91 |
| mondragon | perianth | — | — | — | — | — | — | — | — | 0.025 | 0.005 | 0.03 |
|  | corona | 0.02 | 0.03 | 0.04 | 0.01 | — | — | — | — | 0.23 | 0.04 | 0.37 |
| mount-hood | perianth | — | — | — | — | — | — | — | — | — | — | 0.00 |
|  | corona | 0.003 | 0.007 | — | — | — | — | — | — | — | — | 0.01 |
| pink-charm | perianth | — | — | — | — | — | — | — | — | — | — | 0.00 |
|  | corona | — | — | — | — | — | — | — | — | 0.051 | 0.009 | 0.06 |
| pinza-mutation | perianth | 0.02 | 0.09 | 0.05 | 0.01 | 0.02 | 0.18 | — | — | 0.11 | 0.02 | 0.50 |
|  | corona | 0.09 | 0.17 | 0.09 | 0.02 | 0.1 | 0.21 | 0.05 | 0.06 | 0.12 | 0.04 | 0.95 |
| shangnong-dieying | perianth | 0.003 | 0.005 | — | — | 0.003 | 0.002 | — | — | 0.021 | 0.006 | 0.04 |
|  | corona | 0.09 | 0.18 | 0.08 | 0.03 | 0.07 | 0.1 | — | 0.06 | 0.17 | 0.04 | 0.72 |
| shangnong-ruhuang | perianth | — | — | 0.008 | 0.004 | — | — | — | — | 0.015 | 0.003 | 0.03 |
|  | corona | 0.04 | 0.07 | 0.03 | 0.02 | 0.04 | 0.05 | 0.02 | 0.02 | 0.16 | 0.02 | 0.47 |
| slim-whitman | perianth | — | — | — | — | — | 0.003 | — | — | 0.017 | — | 0.02 |
|  | corona | 0.02 | 0.045 | 0.023 | 0.002 | 0.02 | 0.039 | 0.005 | 0.003 | 0.018 | 0.005 | 0.18 |
| spellbinder | perianth | 0.02 | 0.05 | 0.019 | 0.01 | 0.032 | 0.05 | — | — | 0.021 | 0.008 | 0.21 |
|  | corona | 0.016 | 0.03 | 0.02 | 0.009 | — | — | — | — | 0.015 | 0.01 | 0.10 |
| valdrome | perianth | 0.002 | 0.005 | 0.003 | 0.001 | 0.001 | 0.001 | — | — | 0.007 | — | 0.02 |
|  | corona | 0.04 | 0.08 | 0.07 | 0.03 | 0.02 | 0.11 | — | 0.02 | 0.06 | — | 0.43 |

*^a^*Carotenoid compounds detected in narcissus cultivars; C1: all-*trans*-neoxanthin; C2: 9-*cis*-neoxanthin; C3: all-*trans*-violaxanthin; C4: all-*trans*-antheraxanthin; C5: 9-*cis*- violaxanthin; C6: all-*trans*-lutein; C7: all-*trans*-zeaxanthin; C8: all-*trans*-β-cryptoxanthin; C9: all-*trans*-β-carotene; C10: 9-*cis*-β-carotene.

*^b^*—: Carotenoid compounds didn’t exist or under the detection line.
