# Supplementary material for: Composition of Carotenoids and Flavonoids in Narcissus Cultivars and their Relationship with Flower Color
Source: PLoS One. 2015 Nov 4;10(11):e0142074. doi: 10.1371/journal.pone.0142074 (PMC4633037; doi:10.1371/journal.pone.0142074)
Supplement: S2 Table — (DOCX) [file pone.0142074.s002.docx]

**S2** **Table** **The mean content (mg/g) of flavonoid compounds in perianths and coronas of fifteen narcissus cultivars.**

| Sample | Part | F1*^a^* | F2 | F3 | F4 | F5 | F6 | F7 | F8 | F9 | F10 | F11 | F12 | F13 | F14 | F15 | F16 | F17 | F18 | TF(mg/g) |
| --- | --- | --- | --- | --- | --- | --- | --- | --- | --- | --- | --- | --- | --- | --- | --- | --- | --- | --- | --- | --- |
| avalon | perianth | 0.75 | 0.55 | 0.03 | 0.07 | 0.37 | 0.25 | 0.65 | 0.47 | 0.58 | 0.52 | 0.27 | 0.25 | 0.39 | 0.49 | —*^b^* | 1.21 | 1.27 | 0.92 | 9.04 |
|  | corona | 0.51 | 0.37 | 0.07 | 0.03 | 0.28 | 0.26 | 0.50 | 0.41 | 0.54 | 0.68 | 0.26 | 0.34 | 0.04 | 0.24 |  | 0.27 | 0.26 | 0.47 | 5.63 |
| decoy | perianth | 0.51 | 1.12 | 0.33 | 0.27 | 0.84 | 1.44 | 2.68 | 2.29 | 0.88 | 0.39 | 0.27 | 0.38 | 0.44 | — | — | 0.63 | 1.33 | — | 13.80 |
|  | corona | 0.17 | 0.24 | 0.16 | 0.15 | 0.6 | 0.64 | 1.65 | 1.16 | 0.73 | 0.46 | 0.25 | 0.63 | 0.17 | — | — | 0.36 | 0.33 | 0.2 | 7.74 |
| gigantic-star-mutation | perianth | 0.77 | 0.72 | 0.08 | 0.2 | 0.09 | 0.18 | 1.14 | 0.6 | 1 | 0.53 | 0.08 | 0.21 | 0.26 | 0.27 | — | 1.06 | 0.95 | 0.65 | 9.19 |
|  | corona | 0.74 | 0.54 | 0.05 | 0.11 | 0.08 | 0.63 | 0.47 | 0.63 | 0.29 | 0.04 | 0.11 | 0.15 | 0.15 | 0.74 | — | 0.5 | 0.62 | 0.25 | 5.36 |
| jack-snipe | perianth | 1.14 | 1.38 | 0.56 | 0.62 | 0.26 | 1.56 | 2.14 | 2.32 | 1.48 | 0.88 | 0.82 | 0.58 | 0.15 | — | — | 0.98 | 0.38 | — | 15.37 |
|  | corona | 0.59 | 0.99 | 0.25 | 0.23 | 0.27 | 0.61 | 0.81 | 1.53 | 0.63 | 0.78 | 0.54 | — | — | — | — | 0.14 | 0.54 | — | 7.91 |
| lemon-beauty | perianth | 0.46 | — | — | — | 2.25 | 1.44 | 0.95 | 0.68 | 1.19 | 1.88 | — | — | — | 0.49 | 0.49 | — | 0.30 | — | 10.13 |
|  | corona | 0.44 | — | — | — | 1.68 | 1.06 | 0.93 | 0.56 | 1.12 | 1.99 | — | — | — | 0.41 | 0.4 | — | 0.38 | — | 9.03 |
| marieke | perianth | 0.84 | 0.6 | 0.17 | 0.09 | 0.21 | — | 1.92 | 2.41 | 1.13 | 0.44 | — | 0.18 | 0.31 | — | — | 0.63 | 1.37 | 0.16 | 10.46 |
|  | corona | 0.71 | 0.32 | 0.13 | 0.06 | 0.07 | 0.43 | 1.27 | 1.74 | 0.54 | 0.23 | — | 0.06 | 0.18 | — | — | 0.43 | 1.07 | 0.08 | 7.32 |
| mondragon | perianth | 0.33 | 0.6 | 0.1 | 0.13 | 0.35 | 0.46 | 0.43 | 0.29 | 0.59 | 0.61 | 0.14 | 0.33 | 0.06 | 0.19 | — | 0.08 | 0.13 | 0.15 | 4.97 |
|  | corona | 0.36 | 0.64 | 0.1 | 0.12 | 0.41 | 0.66 | 0.64 | 0.41 | 0.53 | 0.74 | 0.2 | — | — | — | — | — | — | — | 5.04 |
| mount-hood | perianth | 0.58 | 0.5 | 0.06 | 0.17 | 0.04 | 0.15 | 0.82 | 0.4 | 0.72 | 0.37 | 0.03 | 0.23 | 0.34 | 0.35 | — | 0.89 | 0.79 | 0.48 | 6.92 |
|  | corona | 0.69 | 0.35 | 0.05 | 0.11 | 0.15 | 0.07 | 0.6 | 0.47 | 0.62 | 0.22 | 0.03 | 0.11 | 0.17 | 0.15 | — | 0.5 | 0.65 | 0.21 | 5.15 |
| pink-charm | perianth | 0.9 | — | 0.55 | 0.77 | — | 1.61 | 0.67 | 1.58 | 1.12 | 0.54 | 1.15 | — | 0.63 | 0.57 | 0.14 | — | 1.03 | 0.49 | 11.75 |
|  | corona | 0.61 | — | 0.59 | 0.78 | — | 0.95 | 0.89 | 0.98 | 1.1 | 0.17 | 0.73 | — | 0.18 | 0.19 | — | — | 0.36 | 0.15 | 7.68 |
| pinza-mutation | perianth | 0.68 | 0.55 | 0.42 | 0.18 | 0.65 | 0.74 | 1.88 | 1.97 | 0.98 | 0.48 | — | 0.21 | 0.6 | — | — | 0.24 | 0.34 | — | 10.02 |
|  | corona | 0.18 | 0.2 | 0.21 | 0.14 | 0.21 | 0.38 | 1 | 1.04 | 0.56 | 0.43 | 0.1 | — | — | — | — | 0.6 | 0.1 | — | 5.15 |
| shangnong-dieying | perianth | 0.63 | 0.5 | 0.23 | 0.11 | 0.43 | 0.26 | 2.39 | 1.84 | 0.71 | 0.28 | 0.29 | 0.27 | 0.41 | — | — | 0.69 | 0.8 | — | 9.84 |
|  | corona | 0.45 | 0.28 | 0.13 | 0.08 | 0.27 | 0.16 | 1.27 | 1.36 | 0.51 | 0.26 | 0.38 | — | — | — | — | 0.12 | 0.17 | — | 5.44 |
| shangnong-ruhuang | perianth | 0.51 | 0.91 | 0.59 | 0.37 | 0.64 | 0.49 | 2.47 | 2.48 | 1.13 | 0.51 | — | 0.13 | 0.29 | — | — | 0.35 | 0.7 | 0.16 | 11.73 |
|  | corona | 0.13 | 0.34 | 0.24 | 0.08 | 0.13 | 0.13 | 0.78 | 1.16 | 0.36 | 0.18 | — | — | — | — | — | — | 0.65 | — | 4.22 |
| slim-whitman | perianth | 0.35 | 0.66 | 0.34 | 0.11 | 0.31 | 0.18 | 1.4 | 1.86 | 0.48 | 0.2 | 0.43 | 0.09 | 0.21 | — | 0.06 | 0.23 | 0.69 | — | 7.6 |
|  | corona | 0.12 | 0.44 | 0.18 | 0.04 | 0.09 | 0.05 | 0.64 | 1.31 | — | 0.34 | 0.03 | — | — | — | — | — | 0.36 | — | 3.59 |
| spellbinder | perianth | 0.91 | 0.76 | 0.21 | 0.27 | 0.1 | 0.09 | 2.72 | 1.9 | 1.52 | — | — | 0.38 | 0.48 | 0.32 | — | 1.35 | 1.53 | 0.66 | 13.2 |
|  | corona | 0.54 | 0.36 | 0.2 | 0.16 | 0.05 | 0.04 | 1.71 | 1.92 | 1.13 | — | — | 0.1 | 0.17 | 0.05 | — | 0.46 | 0.74 | 0.14 | 7.77 |
| valdrome | perianth | 0.56 | 0.74 | 0.09 | 0.17 | 0.31 | 0.41 | 1.15 | 0.53 | 0.87 | 0.58 | — | 0.24 | 0.36 | 0.35 | — | 0.51 | 0.42 | 0.26 | 7.55 |
|  | corona | 0.38 | 0.58 | 0.16 | 0.17 | 0.32 | 0.56 | 0.74 | 0.48 | 0.59 | 1.13 | — | — | — | — | — | 0.06 | 0.07 | 0.12 | 5.36 |

*^a^*Farotenoid compounds detected in narcissus cultivars; F1: chlorogenic acid; F2: quercetin 7-*O*-dihexoside; F3: quercetin 3,7-di-*O*-hexoside; F4: quercetin 7-*O*-hexoside-pentoside; F5: kaempferol 3,7-di-*O*-hexoside; F6: kaempferol 7-*O*-hexoside-pentoside; F7: isorhamnetin 7-*O*-dihexoside; F8: isorhamnetin 3,7-di-*O*-hexoside; F9: isorhamnetin 7-*O*-hexoside-pentoside; F10: isorhamnetin 3-*O*-rutinoside; F11: isorhamnetin 7-*O*-accerylhexoside-hexoside; F12: quercetin 3-*O*-galactoside; F13: quercetin 3-*O*-glucoside; F14: quercetin 3-*O*-pentoside; F15: kaempferol 3-*O*-glucoside; F16: isorhamnetin 3-*O*-galactoside; F17: isorhamnetin 3-*O*- glucoside; F18: isorhamnetin 3-*O*- pentoside. *^b^*—: Farotenoid compounds were inexistent or under the detection lines.
